# Supplementary figures and images for: Clustering fibromyalgia patients: A combination of psychosocial and somatic factors leads to resilient coping in a subgroup of fibromyalgia patients
Source: PLoS One. 2020 Dec 28;15(12):e0243806. doi: 10.1371/journal.pone.0243806 (PMC7769259; doi:10.1371/journal.pone.0243806)

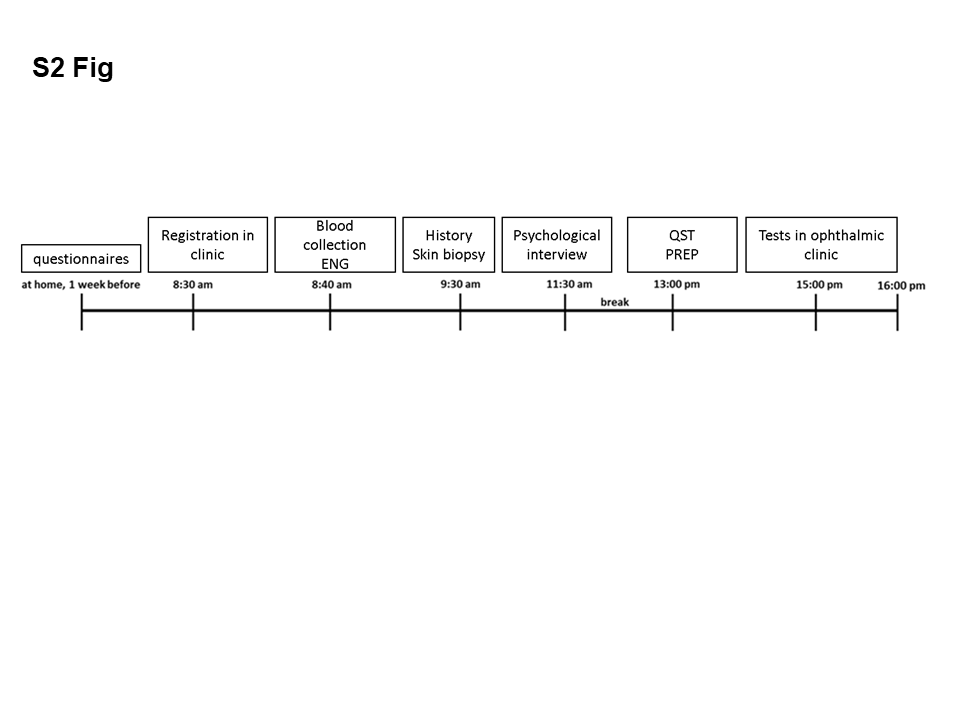

Supplement: S1 Fig — (TIF) [file pone.0243806.s001.tif]

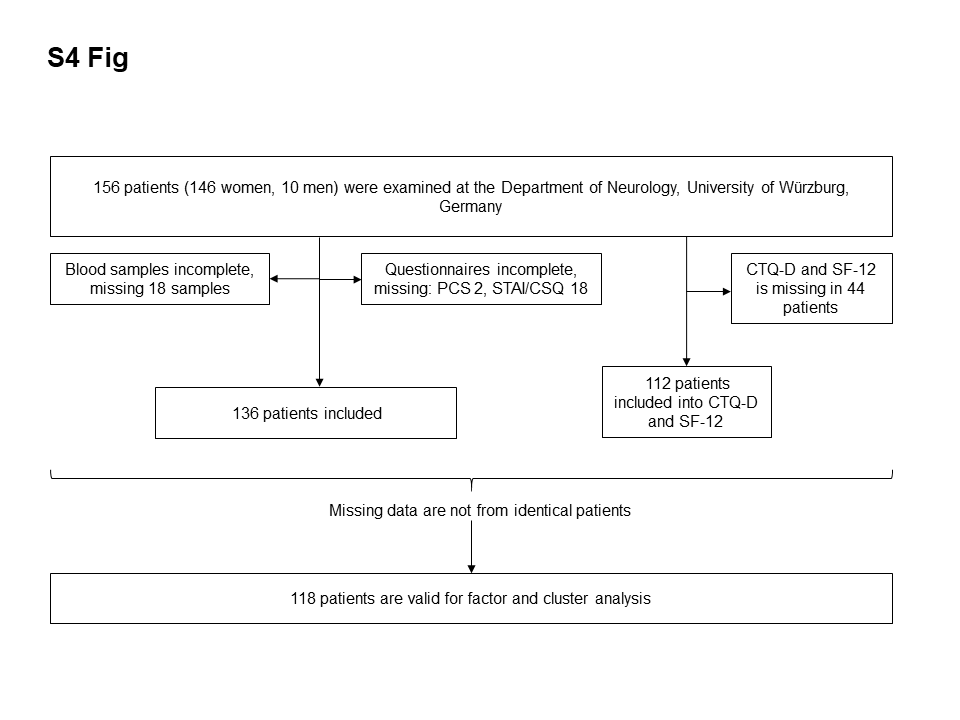

Supplement: S2 Fig — (TIF) [file pone.0243806.s002.tif]

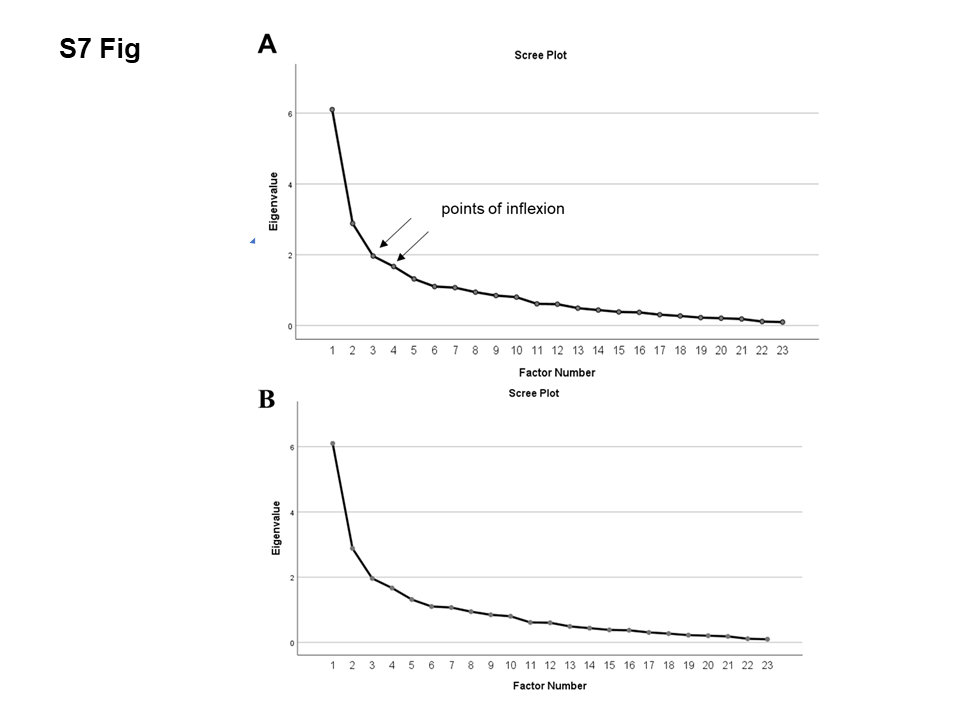

Supplement: S3 Fig — (TIF) [file pone.0243806.s003.tif]

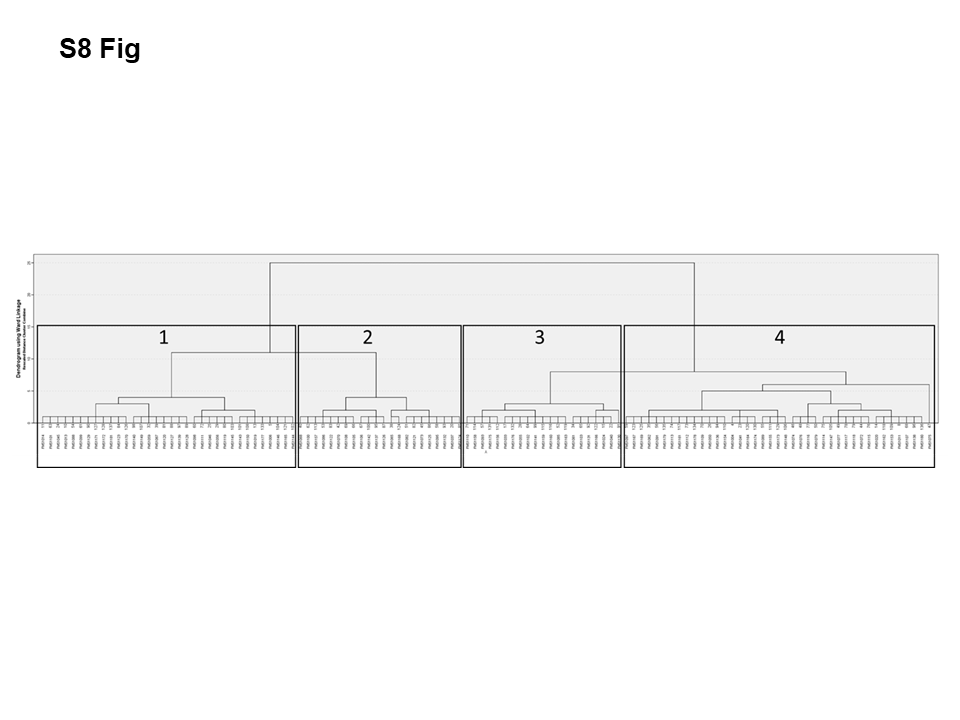

Supplement: S4 Fig — (TIF) [file pone.0243806.s004.tif]
