# Supplementary material for: Clustering fibromyalgia patients: A combination of psychosocial and somatic factors leads to resilient coping in a subgroup of fibromyalgia patients
Source: PLoS One. 2020 Dec 28;15(12):e0243806. doi: 10.1371/journal.pone.0243806 (PMC7769259; doi:10.1371/journal.pone.0243806)
Supplement: S1 Table — (DOCX) [file pone.0243806.s005.docx]

**S1 Table. Exclusion and inclusion criteria of patient recruitment.**

| inclusion criteria |
| --- |
| - male and female patients - at least 18 years old - medically confirmed diagnosis of fibromyalgia syndrome according to the ACR criteria of 1990 and 2010 - willingness to participate in all tests during the study and to travel to the neurological clinic in Würzburg |
| exclusion criteria |
| - other possible differential diagnoses excluded explaining the pain (e.g. rheumatologic, orthopedic) - other and additional pain sources (e.g. pain due to arthritis) - abnormalities in routine blood tests - diabetes - polyneuropathy - ongoing legal proceedings (e.g. regarding health assurance) - psychiatric diseases - cancer (in the last 5 years) - permanent wearing of hard contact lenses - eye surgery / eye diseases - allergies to local narcotics - drug or alcohol abuse - epilepsy - pacemaker |
