# Supplementary material for: Clustering fibromyalgia patients: A combination of psychosocial and somatic factors leads to resilient coping in a subgroup of fibromyalgia patients
Source: PLoS One. 2020 Dec 28;15(12):e0243806. doi: 10.1371/journal.pone.0243806 (PMC7769259; doi:10.1371/journal.pone.0243806)
Supplement: S2 Table — (DOCX) [file pone.0243806.s006.docx]

**S2 Table. Sociodemographic, electrophysiological, laboratory, psychosocial, and somatic characteristic differences among cluster.**

| **characteristics** | **maladaptive**  **cluster A** | | **adaptive**  **cluster B** | | **vulnerable**  **cluster C** | | **resilient**  **cluster D** | |
| --- | --- | --- | --- | --- | --- | --- | --- | --- |
|  | **M^a^** | **SD^b^** | **M^a^** | **SD^b^** | **M^a^** | **SD^b^** | **M^a^** | **SD^b^** |
| **N^c^** | **35** |  | **40** |  | **22** |  | **21** |  |
| **Sociodemographic data** |  |  |  |  |  |  |  |  |
| **gender^d^** | 3^♂^/ 38^♀^ |  | 4^♂^/ 30^♀^ |  | 20^♀^ |  | 22^♀^ |  |
| **age [years]** | 52.1 | 10.0 | 52.4 | 9.6 | 48.5 | 9.6 | 50.0 | 6.4 |
| **Weight [kg]^e^** | 69.8 | 10.6 | 66.3 | 9.8 | 75.8 | 14.3 | 75.5 | 15.7 |
| **Height [cm]^f^** | 163.0 | 5.0 | 164.4 | 6.6 | 168.1 | 3.9 | 164.9 | 7.9 |
| **BMI^g^** | 23.6 | 3.6 | 22.4 | 3.3 | 25.6 | 4.8 | 25.5 | 5.3 |
| **Highest graduation** |  |  |  |  |  |  |  |  |
| Lower secondary school | 7 |  | 6 |  | 6 |  | 3 |  |
| Secondary school | 19 |  | 20 |  | 12 |  | 14 |  |
| High school | 5 |  | 7 |  | 2 |  | 3 |  |
| University | 4 |  | 7 |  | 2 |  | 1 |  |
| **Employment status [N]^c^** |  |  |  |  |  |  |  |  |
| Regularly working | 15 |  | 27 |  | 7 |  | 10 |  |
| Sick leave | 3 |  | 2 |  |  |  | 2 |  |
| Sick leave because of pain | 3 |  | 2 |  | 4 |  | 3 |  |
| Retired | 5 |  | 6 |  | 3 |  | 1 |  |
| Retired because of pain | 5 |  | 1 |  | 5 |  | 5 |  |
| Unemployed | 4 |  | 1 |  | 3 |  |  |  |
| **Time since diagnosis [years]** | 6.4 | 4.6 | 5.4 | 5.1 | 3.9 | 3.0 | 5.5 | 4.5 |
| **Duration of pain due to the disease [years]** | 14.0^*^ | 38.0^*^ | 6.0^*^ | 25.0^*^ | 12.0^*^ | 45.0^*^ | 11.0^*^ | 42.0^*^ |
| **Smoker [N]^c^** | 6 |  | 5 |  | 6 |  | 3 |  |
| **Family history [N]^c^** |  |  |  |  |  |  |  |  |
| Chronic pain | 13 |  | 22 |  | 11 |  | 9 |  |
| Neurological disorder | 4 |  | 5 |  | 7 |  | 5 |  |
| Affective disorder | 8 |  | 3 |  | 3 |  | 3 |  |
| **Psychological/psychiatric treatment [N]^c^** |  |  |  |  |  |  |  |  |
| Never | 12 |  | 18 |  | 6 |  | 8 |  |
| Currently | 11 |  | 12 |  | 14 |  | 7 |  |
| In the past | 12 |  | 10 |  | 2 |  | 6 |  |
| **pain duration [years]** | 14.0^*^ | 38.0^*^ | 6.0^*^ | 25.0^*^ | 12.0^*^ | 45.0^*^ | 11.0^*^ | 42.0^*^ |
| **Questionnaire data** |  |  |  |  |  |  |  |  |
| **NPSI-D sum score^h^** | 0.4^*^ | 0.7^*^ | 0.3 | 0.1 | 0.4 | 0.2 | 0.5 | 0.1 |
| **GCPS-D pain intensity^i^** | 74.4 | 8.8 | 54.3 | 9.1 | 73.4 | 8.1 | 70.5 | 7.9 |
| **GCPS-D disability^i^** | 61.4 | 16.0 | 39.5 | 16.1 | 69.2 | 14.7 | 54.5 | 19.0 |
| **GCPS-D grade^i^** | 2.0^*^ | 3.0^*^ | 1.0^*^ | 1.0^*^ | 2.0^*^ | 2.0^*^ | 2.0^*^ | 2.0^*^ |
| **PCS-D sum score^j^** | 20.9 | 6.9 | 17.9 | 9.7 | 35.6 | 8.0 | 13.0^*^ | 31.0^*^ |
| **CES-D sum score^k^** | 23.1 | 7.1 | 16.0 | 8.7 | 36.6 | 8.8 | 19.0^*^ | 38.0^*^ |
| **FIQ-D sum score^l^** | 48.2 | 8.1 | 36.6 | 9.8 | 57.4 | 8.4 | 50.5 | 8.8 |
| **STAI-S sum score^m^** | 48.2 | 10.2 | 38.0^*^ | 38.0^*^ | 64.6 | 9.1 | 42.0 | 9.8 |
| **STAI-T sum score^m^** | 48.1 | 9.0 | 43.4 | 9.7 | 62.9 | 7.6 | 42.2 | 11.4 |
| **CSQ-D distraction^n^** | 13.5 | 6.2 | 17.0 | 5.4 | 9.1 | 5.3 | 22.0 | 7.2 |
| **CSQ-D reinterpretation^n^** | 3.5^*^ | 21.0^*^ | 4.0^*^ | 22.0^*^ | 1.0^*^ | 9.0^*^ | 10.5 | 7.4 |
| **CSQ-D self instructions^n^** | 20.0^*^ | 32.0^*^ | 23.0^*^ | 25.0^*^ | 17.2 | 6.1 | 27.8 | 3.4 |
| **CSQ-D ignore^n^** | 13.3 | 6.5 | 15.8 | 7.4 | 11.1 | 5.8 | 24.0^*^ | 17.0^*^ |
| **CSQ-D hoping praying^n^** | 7.9 | 4.6 | 9.6 | 4.6 | 9.0 | 6.9 | 15.0^*^ | 21.0^*^ |
| **CSQ-D catastrophizing^n^** | 16.2 | 6.1 | 14.4 | 7.4 | 25.9 | 5.0 | 12.9 | 9.1 |
| **CSQ-D activity increase^n^** | 18.0^*^ | 25.0^*^ | 19.4 | 4.6 | 14.8 | 3.9 | 21.5 | 5.5 |
| **CSQ-D pain behavior^n^** | 19.5^*^ | 25.0^*^ | 20.9 | 4.7 | 18.5 | 32.0 | 19.6 | 5.0 |
| **CSQ-D pain control^n^** | 2.1 | 1.3 | 3.0^*^ | 3.0^*^ | 2.0^*^ | 3.0^*^ | 4.0^*^ | 5.0^*^ |
| **CSQ-D pain reduction^n^** | 2.0^*^ | 4.0^*^ | 3.0^*^ | 3.0^*^ | 2.0^*^ | 4.0^*^ | 2.0^*^ | 4.0^*^ |
| **CTQ-D emotional neglect^o^** | 8.0^*^ | 18.0^*^ | 7.0^*^ | 14.0^*^ | 13.8 | 6.1 | 12.8 | 5.6 |
| **CTQ-D sexual abuse^o^** | 6.0^*^ | 6.0^*^ | 5.0^*^ | 15.0^*^ | 7.5^*^ | 16.0^*^ | 9.3 | 4.2 |
| **CTQ-D physical abuse^o^** | 5.0^*^ | 22.0^*^ | 5.0^*^ | 1.0^*^ | 5.0^*^ | 20.0^*^ | 8.8^*^ | 6.5^*^ |
| **CTQ-D emotional abuse^o^** | 10.9 | 4.8 | 10.4 | 3.9 | 18.0 | 6.0 | 14.7 | 5.5 |
| **CTQ-D physical neglect^o^** | 7.0 | 2.4 | 6.0^*^ | 5.0^*^ | 11.6 | 5.0 | 10.2 | 3.7 |
| **CTQ-D trivialization^o^** | 0.0^*^ | 3.0^*^ | 0.0^*^ | 3.0^*^ | 0.0^*^ | 1.0^*^ | 0.0^*^ | 2.0^*^ |
| **PCR results of cytokines** |  |  |  |  |  |  |  |  |
| **rel. gene expression IL6^p^** | 2.0^*^ | 1.2^*^ | 2.3^*^ | 8.7^*^ | 1.0^*^ | 5.9^*^ | 2.0^*^ | 1.2^*^ |
| **rel. gene expression TNF^p^** | 1.2^*^ | 11.7^*^ | 1.6^*^ | 6.5^*^ | 0.5^*^ | 3.4^*^ | 1.4^*^ | 4.9^*^ |
| **rel. gene expression IL4^p^** | 1.5 | 9.0 | 0.9^*^ | 3.8^*^ | 2.1^*^ | 7.2^*^ | 0.7 | 5.2 |
| **rel. gene expression IL10^p^** | 1.1^*^ | 5.2^*^ | 0.6^*^ | 4.3^*^ | 0.6^*^ | 3.9^*^ | 0.6^*^ | 3.4^*^ |
| **Laboratory measurements** |  |  |  |  |  |  |  |  |
| **HbA1c [%]^q^** | 5.4 | 0.3 | 5.3 | 0.2 | 5.4 | 0.5 | 5.5 | 0.5 |
| **oGGT^r^** |  |  |  |  |  |  |  |  |
| Before oGGT [mg %] | 95.6 | 11.2 | 93.2 | 8.8 | 100 | 0.19 | 98.3 | 8.8 |
| oGGT (1h value) [mg %] | 148.1 | 39.4 | 153.7 | 38.1 | 146.7 | 37.8 | 145.8 | 28.3 |
| oGGT (2h value) [mg %] | 127.0 | 31.3 | 116.9 | 19.6 | 124.9 | 23.7 | 119.9 | 15.6 |
| **Vitamin B12 [pg/ml]** | 449.5 | 140.5 | 536.6 | 206.5 | 560.9 | 391 | 554.7 | 242 |
| **TSH [mlU/l]^s^** | 3.2 | 8.9 | 1.7 | 1.3 | 1.6 | 0.8 | 2.1 | 1.4 |
| **Vitamin D [µg/l]** | 27.5 | 8.9 | 33.6 | 10.7 | 25.6 | 7.1 | 26.2 | 9.9 |
| **Clinical examination** |  |  |  |  |  |  |  |  |
| **Pain character [%]** |  |  |  |  |  |  |  |  |
| Tearing | 3 |  | 4 |  | 2 |  | 2 |  |
| Pressing | 12 |  | 12 |  | 13 |  | 13 |  |
| Burning | 19 |  | 13 |  | 11 |  | 4 |  |
| Muscle sourness | 9 |  | 12 |  | 4 |  | 5 |  |
| Stabbing | 12 |  | 9 |  | 3 |  | 5 |  |
| **Pain distribution type** |  |  |  |  |  |  |  |  |
| Proximal | 10 |  | 10 |  | 7 |  | 3 |  |
| Distal | 1 |  | 0 |  | 2 |  | 0 |  |
| Whole body | 23 |  | 30 |  | 11 |  | 17 |  |
| **Paresthesia** |  |  |  |  |  |  |  |  |
| **Current pain intensity [NRS scale 0 - 10]^t^** | 6.0 | 1.6 | 4.2 | 1.5 | 5.8 | 1.8 | 5.6 | 1.8 |
| **Electrophysiological data - QST^u^** |  |  |  |  |  |  |  |  |
| CDT | -2.7 | 2.1 | -2.9 | 4.0 | -4.2 | 2.7 | -4.0 | 2.7 |
| WDT | 6.6 | 3.8 | 7.6 | 3.8 | 7.2 | 3.7 | 7.8 | 2.4 |
| TSL | 10.6 | 7.2 | 12.0 | 6.8 | 11.8 | 5.6 | 12.0 | 4.5 |
| PHS | 0.4 | 1.0 | 0.4 | 1.0 | 0.7 | 1.1 | 0.0 | 0.8 |
| CPT | 18.3 | 7.6 | 17.9 | 6.6 | 16.3 | 6.1 | 16.2 | 7.2 |
| HPT | 44.1 | 3.3 | 45.7 | 2.9 | 44.8 | 2.9 | 46.0 | 1.9 |
| MDT | 3.9 | 4.8 | 3.9 | 3.8 | 4.0 | 4.5 | 2.1 | 3.5 |
| MPT | 62.3 | 58.5 | 151.4 | 156.6 | 63.9 | 111.3 | 74.5 | 59.1 |
| MPS | 5.0 | 9.5 | 3.9 | 5.4 | 4.4 | 4.7 | 3.7 | 5.5 |
| DMA | 0.5 | 2.0 | 0.0 | 0.1 | 0.3 | 1.1 | 0.0 | 0.0 |
| WUR | 2.9 | 1.7 | 3.1 | 1.8 | 2.8 | 1.5 | 3.7 | 3.1 |
| VDT | 6.0 | 1.1 | 6.4 | 0.9 | 6.4 | 1.2 | 6.3 | 1.4 |
| PPT | 336.2 | 87.9 | 384.1 | 171.9 | 380.6 | 157.6 | 477.7 | 133.7 |
| **PREP^v^** |  |  |  |  |  |  |  |  |
| Face N1 [ms] | 137.0 | 10.3 | 135.2 | 12.4 | 136.2 | 14.5 | 121.4 | 36.4 |
| Face P1 [ms] | 184.9 | 10.7 | 183.3 | 12.6 | 178.6 | 18.1 | 161.4 | 49.8 |
| Face PPA [mV] | - | - | - | - | - | - | - | - |
| Foot N1 [ms] | 144.9 | 57.9 | 132.5 | 55.7 | 143.3 | 61.4 | 118.4 | 66.7 |
| Foot P1 [ms] | 182.7 | 73.4 | 162.1 | 66.3 | 179.1 | 76.1 | 149.1 | 88.4 |
| Foot PPA [mV] | - | - | - | - | - | - | - | - |
| NFD [no/mm2] | 22.1 | 6.8 | 25.9 | 6.2 | 23.5 | 6.6 | 22.7 | 7.4 |
| NBD [no/mm2] | 64.8 | 34.7 | 76.4 | 40.3 | 85.1 | 38.1 | 77.7 | 32.8 |
| NFL [mm/mm2] | 12.8 | 3.6 | 14.2 | 3.1 | 13.8 | 3.5 | 13.6 | 3.7 |
| **skin biopsy^w^** |  |  |  |  |  |  |  |  |
| IENFD lower leg [fibers/mm] | 8.5 | 3.5 | 9.2 | 3.4 | 8.2 | 3.4 | 7.4 | 3.0 |
| IENFD upper thigh [fibers/mm] | 7.1 | 2.7 | 7.8 | 3.1 | 6.4 | 2.7 | 5.7 | 3.6 |

*^a^ M = mean; ^b^ SD = standard deviation; ^c^ N = number; ^d^gender: ^♂^: men; ^♀^: women; ^e^weight in kg = kilogram; ^f^height in cm = centimetre; ^g^BMI = body mass index; ^h^NPSI-D = German version of the neuropathic pain scale inventory; ^i^ GCPS-D = three subscales of the German version of the graded chronic pain scale; ^j^PCS = German version of the pain catastrophizing scale; ^k^ CES-D = German version of the center of epidemiological studies general depression scale; ^l^FIQ-D = German version of the fibromyalgia impact questionnaire; ^m^subscales trait (T) and state (S) of the German version of the state/trait anxiety inventory (STAI-G); ^n^ CSQ-D = ten subscale scores of the German version of the coping strategies questionnaire; ^o^CTQ-D = six subscale scores of the German version of the childhood trauma questionnaire; ^p^relative gene expression values of four cytokines: IL = interleukin, TNF = tumor necrosis factor; ^q^HbA1c = glycosylated haemoglobin; ^r^oGTT = oral glucose tolerance test; ^s^TSH = thyroid-stimulating hormone; ^t^NRS = numeric rating scale; ^u^ twelve values of QST (= quantitative sensory testing): CDT = cold detection threshold, WDT = warm detection threshold, TSL = capability to identify temperature alterations, thermal sensory limen, PHS = paradoxical heat sensation, CPT = cold pain threshold, HPT = heat pain threshold, MDT = mechanical detection threshold, MPS = mechanical pain sensitivity, DMA = dynamic mechanical allodynia, WUR = wind up ratio, VDT = vibration detection threshold, PPT = pressure pain threshold*

*^v^three values for PREP (= pain-related evoked potential measurements), each for face and foot:*

*N1 = first negative peak, P1 = first positive peak, PPA = peak-to-peak amplitudes; ^w^IENFD = intraepidermal nerve fibre density (derived from skin biopsies of lower and upper leg).*

*^*^All not normally distributed data are given as median (MED) and range (R) respectively.*
