# Supplementary material for: Clustering fibromyalgia patients: A combination of psychosocial and somatic factors leads to resilient coping in a subgroup of fibromyalgia patients
Source: PLoS One. 2020 Dec 28;15(12):e0243806. doi: 10.1371/journal.pone.0243806 (PMC7769259; doi:10.1371/journal.pone.0243806)
Supplement: S3 Table — (DOCX) [file pone.0243806.s007.docx]

**S3 Table. Adequacy tests of the principal axis factoring analysis.**

| **Kaiser-Meyer-Olkin (KMO) measure of sampling adequacy** |  | 0.8 |
| --- | --- | --- |
| **Bartlett’s test of sphericity** | **chi^2^** | 1322.3 |
|  | **df^a^** | 253 |
|  | **p^b^** | 0.0 |

*^a^df = degree of freedom; ^b^p value of significance*
