# Supplementary material for: Clustering fibromyalgia patients: A combination of psychosocial and somatic factors leads to resilient coping in a subgroup of fibromyalgia patients
Source: PLoS One. 2020 Dec 28;15(12):e0243806. doi: 10.1371/journal.pone.0243806 (PMC7769259; doi:10.1371/journal.pone.0243806)
Supplement: S4 Table — (DOCX) [file pone.0243806.s008.docx]

**S4 Table. Data of variance of emerged factors with eigenvalues more than 1 observed between predicting variables.**

|  |  | **initial Eigenvalues** |  |
| --- | --- | --- | --- |
| **factor** | **total** | **% of variance** | **cumulative %** |
| **1** | 6.1 | 26.5 | 26.5 |
| **2** | 2.9 | 12.5 | 39.1 |
| **3** | 2.0 | 8.5 | 47.6 |
| **4** | 1.7 | 7.3 | 54.9 |
| **5** | 1.3 | 5.7 | 60.6 |
| **6** | 1.1 | 4.8 | 65.4 |
| **7** | 1.1 | 4.7 | 70.0 |

*Extraction method: principal axis factoring.*
