# Supplementary material for: Clustering fibromyalgia patients: A combination of psychosocial and somatic factors leads to resilient coping in a subgroup of fibromyalgia patients
Source: PLoS One. 2020 Dec 28;15(12):e0243806. doi: 10.1371/journal.pone.0243806 (PMC7769259; doi:10.1371/journal.pone.0243806)
Supplement: S5 Table — (DOCX) [file pone.0243806.s009.docx]

**S5 Table. One-way ANOVA to test the significance between factors between the subgroups.**

| **factor** |  | **sum of squares** | **df^a^** | **mean square** | **F^b^** | **p^c^** |
| --- | --- | --- | --- | --- | --- | --- |
| **affective load** | between groups | 55.1 | 3 | 18.4 | 38.1 | 0.001^d^ |
|  | within groups | 54.9 | 114 | 0.5 |  |  |
|  | total | 110.1 | 117 |  |  |  |
| **coping** | between groups | 48.3 | 3 | 16.1 | 33.9 | 0.001^d^ |
|  | within groups | 54.1 | 114 | 0.5 |  |  |
|  | total | 102.4 | 117 |  |  |  |
| **Physical functioning** | between groups | 55.2 | 3 | 18.4 | 55.9 | 0.001^d^ |
|  | within groups | 37.6 | 114 | 0.3 |  |  |
|  | total | 92.8 | 117 |  |  |  |
| **pro-inflammatory cytokines** | between groups | 5.9 | 3 | 2.0 | 3.4 | 0.05^e^ |
|  | within groups | 66.8 | 114 | 0.6 |  |  |
|  | total | 72.7 | 117 |  |  |  |

*^a^df = degree of freedom; ^b^F = value of test statistic ; ^c^p = level of significance; ^d^Level of significance is p < 0.001; ^e^level of significance is p < 0.05.*
