# Supplementary material for: Clustering fibromyalgia patients: A combination of psychosocial and somatic factors leads to resilient coping in a subgroup of fibromyalgia patients
Source: PLoS One. 2020 Dec 28;15(12):e0243806. doi: 10.1371/journal.pone.0243806 (PMC7769259; doi:10.1371/journal.pone.0243806)
Supplement: S6 Table — (DOCX) [file pone.0243806.s010.docx]

**S6 Table. Post-hoc analysis between subgroups and factors.**

| **Cluster** | | **Games - Howell, * p < 0.05** | | | |
| --- | --- | --- | --- | --- | --- |
| **A** | **B** | * |  | * | * |
|  | **C** | * | * |  |  |
|  | **D** | * | * |  | * |
| **B** | **A** | * |  | * |  |
|  | **C** | * | * | * |  |
|  | **D** |  | * | * |  |
| **C** | **A** | * | * |  |  |
|  | **B** | * | * | * |  |
|  | **D** | * | * |  |  |
| **D** | **A** | * | * |  |  |
|  | **B** |  | * | * |  |
|  | **C** | * | * |  |  |
| **factor** | | **affective load** | **coping** | **physical functioning** | **pro-inflammatory cytokines** |

** significant differences are marked.*
